# GRP78 PROMOTES THE OSTEOGENIC AND ANGIOGENIC RESPONSE IN PERIODONTAL LIGAMENT STEM CELLS

**DOI:** 10.22203/eCM.v045a02

**Published:** 2023-01-23

**Authors:** A. Merkel, Y. Chen, C. Villani, A. George

**Affiliations:** Department of Oral Biology, University of Illinois at Chicago, Chicago, Illinois 60612, USA

**Keywords:** Stem cells, tissue engineering, periodontal disease, GRP78, osteoblastogenesis, angiogenesis

## Abstract

Periodontitis is a progressive disease that ultimately leads to bone and tooth loss. A major consequence of periodontal disease is the inability to regain lost bone in the periodontium. The importance was demonstrated of glucose-regulated protein-78 (GRP78) in the osteogenic differentiation of periodontal ligament stem cells and their potential use for regeneration of the periodontium. Previous studies have shown the relationship between GRP78 and dentine matrix protein-1 (DMP1). The importance of this receptor-ligand complex in supporting the process of osteogenesis and angiogenesis was confirmed in this study. To show the function of GRP78 in mineralised tissues, transgenic periodontal ligament stem cells (PDLSCs) were generated in which *GRP78* was either overexpressed or silenced. Gene expression analysis of the cells cultured under osteogenic conditions showed an increase in key osteogenic genes with the overexpression of *GRP78*. RNA-Seq analysis was also performed to understand the transcriptome profile associated with genotype changes. Using the database for annotation, visualisation, and integration discovery (DAVID) for the functional enrichment analysis of differentially expressed genes, the upregulation of genes promoting osteogenesis and angiogenesis with *GRP78* overexpression was demonstrated. Alizarin red staining and scanning electron microscopy analysis revealed matrix mineralisation with increased calcium deposition in GRP78 overexpressing cells. The *in vivo* osteogenic and angiogenic function of GRP78 was shown using a subcutaneous implantation rodent model. The results suggested that *GRP78* in PDLSCs can regulate the expression of both osteogenesis and angiogenesis. Therefore, GRP78 could be considered as a therapeutic target for repair of diseased periodontium.

## Editor’s note

There were no questions from reviewers for this paper; therefore, there is no Discussion with reviewers section. The Scientific Editor responsible for this paper was Thimios Mitsiadis.

## Introduction

The formation of bone and dentine in vertebrates requires a precise coordination of cells and proteins to assemble a functional mineralised matrix. A healthy periodontium is comprised of the alveolar bone, gingiva, and periodontal ligament (Hughes, 2015). Periodontal disease is widespread, affecting almost half of the adult United States population, and the management for this disease is often surgical therapy and maintenance. Throughout the ageing process, the periodontium can become reduced due to periodontal disease, trauma, tooth loss due to caries, systemic diseases, and extrinsic factors. Regeneration of the periodontium requires activation of the osteogenic and angiogenic processes to form new cellular tissues (Dimitriou et al., 2011). Many researchers have studied various aspects of the regeneration process; however, the regeneration of the periodontium has limitations. It was demonstrated, for the first time, using protein and gene expression analysis that overexpression of GRP78 in periodontal ligament stem cells led to an increase in their differentiation into osteogenic lineage with an upregulation of angiogenesis markers. Additionally, the translational potential was shown, using an *in vivo* subcutaneous implantation rodent model, because of the upregulation of both angiogenic and osteogenic markers. This novel approach revealed the use of GRP78 as a promising therapeutic for periodontium regeneration and repair of tissues lost due to periodontal disease.

DMP1 is a non-collagenous extracellular matrix protein of the SIBLING family that is present in both bone and dentine (He et al., 2003). DMP1 is a vital protein in the development of both bone and dentine due to its multifaceted functions. The protein has a nuclear localisation signal that allows DMP1 transport to the nucleus (Jacob et al., 2014; Narayanan et al., 2003). In the nucleus, DMP1 can function as a transcriptional regulator and aid in the terminal differentiation of pre-osteoblasts and pre-odontoblasts (Narayanan et al., 2003). In the extracellular matrix, DMP1 can act as a nucleation site for hydroxyapatite when bound to the self-assembled collagen fibrils, thus functioning as a regulatory protein in the extracellular matrix of bone and dentine (George et al., 2018. DMP1 binds specifically to type I collagen and regulates mineral nucleation and growth. In Biomineralization, 137-145. Singapore: Springer Singapore. DOI: 10.1007/978-981-13-1002-7_15: Conference abstract). Studies on knock-out DMP1 mouse models show altered structure of the periodontal ligament and alveolar bone (Ren et al., 2016). Although DMP1 was first identified as an extracellular matrix protein, the importance of its nuclear function is not well studied.

Internalisation of DMP1 within the cell is mediated by a receptor protein, GRP78 (Merkel et al., 2019; Ravindran et al., 2012). This protein is a member of the heat-shock family of proteins that work throughout the cell in response to stress. GRP78 is the master regulator of the endoplasmic reticulum in terms of maintaining homeostasis by binding to unfolded proteins and maintaining physiological intracellular calcium concentrations (Lee, 2001). One of its main functions is to regulate the UPR, which is an intracellular regulatory mechanism that is activated in response to stress. When the cell signals a stress response, GRP78 disassociates from downstream ER sensors, ATF6, PERK, and IRE1 to signal through various mediators depending upon the requirement of the cell to restore homeostasis. Each of the 3 pathways leads to various responses such as the transcription of cytoprotective genes, transcription of splicing factors, or a cascade to apoptosis depending on the level of cellular damage (Walter and Ron, 2011; Zhu and Lee, 2015).

Although GRP78 resides in the ER, it can function within various cellular domains like the plasma membrane, ER and the cytoplasm. DMP1 is internalised through its receptor GRP78 that is localised on the plasma membrane in response to stress (Merkel et al., 2019). This receptor-ligand interaction demonstrates the multidimensional functions of GRP78 (Ni et al., 2011). Endocytosis is mediated through the caveolin pathway and assisted by the Rab proteins, which are involved in the cellular trafficking of endosomes. Various Rab proteins coordinate the movement of DMP1 and GRP78 complex from the plasma membrane to the nucleus (Merkel et al., 2019). In the nucleus, DMP1 functions in the differentiation of pre-odontoblasts and pre-osteoblasts to terminally differentiated cells. The interaction between DMP1 and GRP78 demonstrates the importance of these proteins in the differentiation of stem cells and ultimately matrix mineralisation.

The role of GRP78 in matrix mineralisation and vasculogenesis was demonstrated using hPDLSCs with silenced or overexpressed *GRP78*. Understanding the function of GRP78 in PDLSCs will help in the development of a novel cell-based therapy for bone regeneration.

## Materials and Methods

### Cell culture

hPDLSCs were isolated and characterised (Seo et al., 2004). The stem cell marker, STRO-1, was used to confirm stemness (Lv et al., 2014). The hPDLSCs were grown in normal growth media (αMEM, 10 % FBS, 1 % antibiotics, 1 % L-Glutamine). A stable cell line of hPDLSCs-*GRP78* was made by transduction of the pCDH-GRP78-GFP plasmid that was packaged with Lentivirus. The cells were selected with puromycin for 2 weeks. GFP expression was confirmed in 95 % of the cells by light microscopy. Silencing of *GRP78* was performed using packaged sh-GRP78 Lentivirus (Santa Cruz Biotechnology, Dallas, TX, USA) transduced into hPDLSCs followed by selection with puromycin. For experiments to determine osteogenic potential, the hPDLSCs-*GRP78* were cultured in normal growth media supplemented with 10 mmol/L β-glycerophosphate (Thermo Fisher Scientific), 100 μg/mL ascorbic acid (Sigma-Aldrich, St. Louis, MO, United States), and 10 nmol/L dexamethasone (MP Biomedicals, Santa Ana, CA, United States). For experiments requiring DMP1 stimulation, 50 ng/mL of recombinant DMP1 was used.

### Quantitative real time PCR

The three cell types hPDLSCs, hPDLSCs-OE-*GRP78*, and hPDLSCs-sh-*GRP78* were cultured under OD conditions for 1, 2 and 3 weeks. Total RNA was extracted from harvested cells using RNeasy Plus Mini Kit (QIAGEN, Germantown, MO, United States) according to the manufacturer’s protocol. cDNA was synthesised with Superscript III Reverse Transcriptase and Oligo-dT primer (Thermo Fisher Scientific) for 60 min at 50° C. qPCR was carried out using FastStart Universal SYBR Green Master reagent (Roche diagnostics, Indianapolis, IN, United States) using specific primers on an ABI StepOnePlus instrument (Thermo Fisher Scientific). The gene expression levels were estimated using the 2^−ΔΔCT^ method with GAPDH expression at 0 weeks used as the internal control. Primers were synthesised by IDT (Integrated DNA Technologies, Inc., Coralville, IA, United States) (Merkel et al., 2019).

### Immunocytochemistry

hPDLSCs-OE-*GRP78* were seeded on glass coverslips and cultured in normal growth or OD media to 70-80 % confluence prior to treatment with recombinant DMP1, which was expressed as previously described (Chandrasekaran et al., 2013). The hPDLSCs-OE-*GRP78* were stimulated with rDMP1 and analysed at 15 and 30 min. The cells were permeabilised with 0.25 % Triton-X in PBS for 30 min and incubated with anti-DMP1 rabbit polyclonal antibody (Chandrasekeran et al., 2013) and DAPI. Fluorescent Rabbit TRITC secondary antibodies (1/100; Sigma-Aldrich) was used, and the slides were mounted and visualised using a Zeiss 710 Meta Confocal Microscope at the UIC Core Facility. The images were analysed through JACoP ImageJ to determine the PCC with the auto-determined threshold (Bolte and Cordelières, 2006; Rueden et al., 2017).

### Protein isolation and immunoblotting

hPDLSCs-OE-*GRP78* were grown under normal growth and differentiation conditions with or without DMP1 treatment for 24 h. Cells were then harvested and lysed in RIPA buffer (cell signalling) containing protease and phosphate inhibitor cocktail (Millipore). Centrifugation was then performed at 11,500 ×*g* for 15 min at 4° C and the supernatants were used as total cellular proteins. Protein concentrations were measured using the Bio-Rad Protein Assay Dye Reagent (BIO-RAD) with BSA as standard. 25 μg of total proteins were loaded on a 10 % SDS-polyacrylamide gel. The proteins were transferred onto a nitrocellulose membrane following electrophoresis, blocked with 5 % skim milk (Merkel et al., 2019). The membranes were incubated with the following primary antibodies: anti-TRIP-1 rabbit polyclonal antibody (1/1000; Invitrogen), anti-FL DMP1 (1/1000; house-made), anti-DPP rabbit polyclonal antibody (1/1000; house-made) (Eapen et al., 2012). Anti-tubulin mouse monoclonal antibody (1/5000; Invitrogen) was used as a loading control. The blots were incubated in either anti-mouse or anti-rabbit secondary conjugated with HRP. Each of the blots were washed 4 times with PBS, and the bands were visualised using chemiluminescence detection (Thermo Fisher Scientific) using X-ray films according to the manufacturer’s protocol.

### RNA sequencing

Total RNA was extracted from harvested cells using an miRNeasy Plus Mini Kit (QIAGEN, Germantown, MO, United States) according to the manufacturer’s protocol. The hPDLSCs overexpressing GRP78 were grown under normal growth and OD conditions for 2 d. The samples were analysed at the UIC Genomics Core for whole transcript strand-specific mRNA sequencing. The library was constructed, quantified, and sequenced with NextSeq500. This was done at a high output, 2 × 42, 450 M reads/run to achieve at least 25 M clusters/sample.

### Bioinformatics analysis

The data was analysed by the UIC Bioinformatics Core, which consisted of gene and isoform expressions quantification and differential expression. Prior to differential expression analysis, PCA were performed to identify biological outliers that should be removed or further investigated. The differential gene expression was performed with 2-factor multi-group analysis and pairwise analysis to yield log_2_-fold change, log_2_ CPM, and *p*-values for each gene. Normalised log count per million values (from the edgeR differential analysis, in *edgeR*) were filtered to an adjusted *p*-value of 0.05 and z-scored using *R* analysis software (R Core Team in RStudio (RStudio Team) prior to heat map generation (Robinson et al., 2010). Significant genes were determined based on an FDR threshold of 5 % (0.05) for osteogenesis and 1 % for angiogenesis (0.01) in the multi-group comparison. The more stringent cut-off is due to the large number of genes associated with angiogenesis group compared to osteogenesis. The DAVID Software (Web ref. 1) was used to generate heatmaps specifically for angiogenic and osteogenic genes. The data obtained were further analysed using Qiagen IPA software (Qiagen, Hilden, Germany). Pairwise comparisons were matched to the IPA library of canonical pathways. Core Analysis within the IPA Software was performed to understand the relationships, mechanisms, canonical pathways, up/downstream molecules, and interaction networks to develop pathways in order to understand the biological cues behind the large gene datasets. The angiogenesis pathway in IPA came from “HIF1α signalling,” module and the osteogenesis pathway came from “Role of osteoblasts in Rheumatoid Arthritis Signalling Pathway”.

### *In vitro* mineralisation assay and nodule detection by alizarin red staining

To determine the presence of Ca^2+^ in the matrix by alizarin red staining, hPDLSCs, hPDLSCs-OE-*GRP78* and hPDLSCs-sh-*GRP78* were seeded on 6-well plates and cultured in OD media containing 10 mmol/L β-glycerophosphate (Thermo Fisher Scientific), 0.50 mmol/L ascorbic acid (Sigma Aldrich) and 10 nmol/L dexamethasone (Sigma-Aldrich) for 0, 1, 2 and 3 weeks. At each time point, the cells were washed with PBS and fixed in 10 % neutral formalin at 4°C for 4 h. The cells were stained with 2 % alizarin red solution (Sigma-Aldrich) for 30 min, and then rinsed with water. The plates were scanned to visualise the overall staining pattern and high-magnification images were obtained using a light microscope (Zeiss Axio observer D1 phase contrast inverted microscope). For the quantification, the bonded alizarin dye was extracted using 10 % acetic acid solution at 21°C for 1 h. followed by 85° C for 2 h. The mixture was then centrifuged at 8,225 ×*g* for 10 min and the supernatant was collected. Ammonium water (10 %) was added (1/5 volume) to the solution and absorbance at 405 nm was determined using a Bioteck plate reader. Alizarin red was used to generate a standard curve (Gregory et al., 2004).

### SEM

The hPDLSC, hPDLSC-OE-*GRP78*-GFP and hPDLSCs-sh-*GRP78* cells were seeded on a cover glass (12 mm, Invitrogen) and cultured under normal growth medium as described in the previous section until confluent. Cells were then incubated in OD medium and were replaced with fresh osteogenic medium every 2 d until harvest at the indicated time points (1 week and 2 weeks). At each time point, the cells were washed with PBS and subsequently fixed in 10 % neutral buffered formalin for 2 h at room temperature. The cells were washed extensively with water and dehydrated with gradient ethanol, then treated with hexamethyldisilazane (Electron Microscopy Sciences) (Hatfield, PA) and dried. The cells were sputter coated with 10 nm gold/palladium and images obtained using an SEM (JEOL JSM-IT500HR, JEOL USA, Inc., Peabody, MA). Conditions used were as published earlier (Chen et al., 2021).

### *In vivo* assay using a subcutaneous implantation rodent model to determine the osteogenic and angiogenic potential of GRP78

hPDLSCs, hPDLSCs-sh-*GRP78*, and hPDLSCs-OE-*GRP78* were grown until 80 *%* confluency and seeded onto 1 cm^2^ sections of collagen tape (Zimmer, Warsaw, IN, United States) at a density of 1 × 10^6^ cells/scaffold. Cell-seeded scaffolds were cultured *in vitro* for 24 h. The following day, scaffolds with the cells adsorbed were implanted subcutaneously on the dorsum of nude male mice according to UIC protocol Animal Assurance Number 19-001. The three groups with *n* = 4 according to power analysis were: scaffold with hPDLSCs, scaffold with hPDLSCs-sh-*GRP78* and scaffold with hPDLCSs-OE-*GRP78*. Four weeks post implantation, the animals were sacrificed, and the scaffolds were retrieved, fixed in 4 % neutral buffered formalin. They were then paraffin embedded and sectioned into 5 μm thick sections for histological evaluations according to published protocols (Fischer et al., 2008a; Fischer et al., 2008b; Fischer et al., 2008c).

### Histology and immunofluorescence

All sections were dewaxed in xylene, rehydrated in graded ethanol solutions and H&E staining was performed on the sections according to published protocols (Fischer et al., 2008d). Picrosirius red staining was performed on the sections according to published protocols and analysed using polarised light microscopy (Rittié, 2017). For immunofluorescence the processed sections were probed with anti-COL1A1 mouse monoclonal (1/100; Cell Signaling Technology, Danvers, MA), anti-HIF1α rabbit polyclonal antibody (1/100; Santa-Cruz Biotechnology, Dallas, TX, United States), anti-RUNX2 rabbit polyclonal ((1/100; Cell Signaling Technology), anti-GRP78 mouse monoclonal antibody (1/100; Santa-Cruz Biotechnology), anti-DMP1 rabbit polyclonal antibody (1/100; made in house) (Eapen et al., 2012), anti vWF mouse monoclonal (1/100; Cell Signalling Technology,), anti-FN rabbit polyclonal antibody (1/100; Santa-Cruz Biotechnology), anti-OPN rabbit polyclonal antibody (1/100; Santa-Cruz Biotechnology), anti-OCN rabbit polyclonal antibody (1/100; Santa-Cruz Biotechnology), anti-VEGFA mouse monoclonal (1/100; Cell Signalling Technology), anti-VEGFR2, anti-PTX3 mouse monoclonal (1/100; Cell Signalling Technology), and anti-CD31. Fluorescent anti-mouse monoclonal and anti-rabbit polyclonal secondary antibodies were used along with DAP1. Slides were analysed at the UIC Microscopy Core imaging facility. All comparative fluorescence images were obtained using the same imaging conditions. Cellular fluorescence densities were quantified using ImageJ (version 1.53 s) and values were analysed for statistical significance using *ANOVA* with *post-hoc* Tukey HSD Test.

### Statistical analysis

For gene expression from *in vitro* cultured cells, alizarin red quantification, the statistical analysis *p* values were calculated using one-way *ANOVA* followed by Tukey’s multiple comparison test. A statistically significant difference is denoted by * *p* < 0.05 *vs*. control; ** *p* < 001. For both immunohistochemistry and immunocytochemistry, the experiments were performed with *n* ≥ 3 sections. For quantification of the colour intensity, student's *t*-test was used and *p* < 0.05 was considered significant. For RNA sequencing, the experiment was performed with *n* = 3, and the results were analysed through the UIC Bioinformatics Core. For pathway analysis, fold changes less than 2 were not included in the analysis, and all significance values were of *p* ≤ 0.05.

### Data availability statement

Transcriptome data used across the analysis was deposited in the Sequencing Read archive under BioProject accession: PRJNA824064.

## Results

### DMP1 and GRP78 colocalise in hPDLSCs overexpressing GRP78

**Fig. 1.**
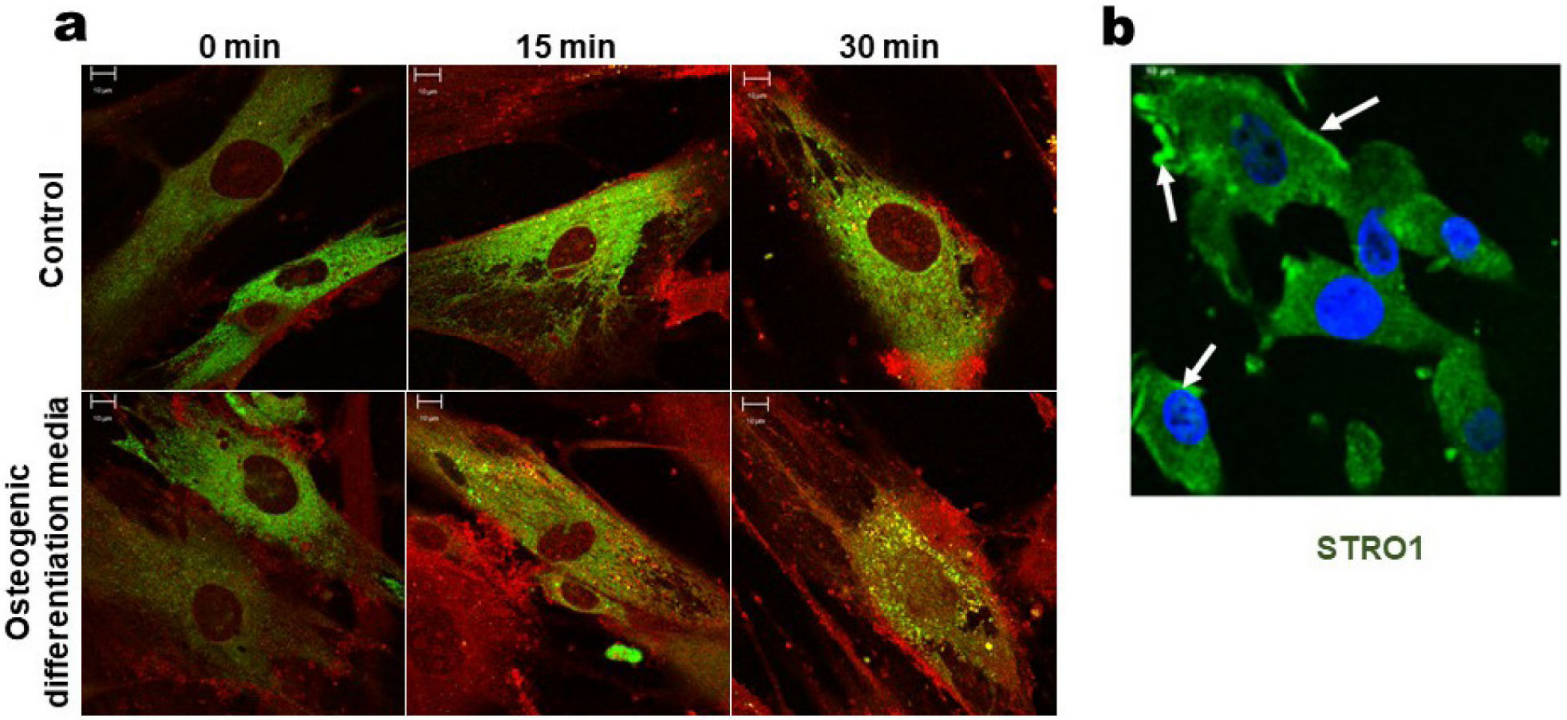
Localisation of GRP78 and DMP1 in hPDLSCs overexpressing GRP78. (**a**) PDLSCs overexpressing GRP78 were stimulated with DMP1 for 15 and 30 min and examined for colocalisation in normal growth and osteogenic media. Confocal imaging demonstrate immunolocalisation of GRP78 (GFP) and DMP1 (TRITC) in normal growth (control) and OD media. Note higher colocalisation with OD conditions. Scale bars = 10 μm. (**b**) Immunofluorescence of hPDLSCs with stem cell marker STRO-1 (GFP). Arrows represent membrane localisation. Scale bars = 10 μm.

Colocalisation of GRP78 and DMP1 was observed in cells overexpressing GRP78 in both control growth and OD media as indicated in yellow in Fig. 1a. Upon stimulation with rDMP1, colocalisation could be observed near the plasma membrane in control growth media as shown by the arrow at 15 min with a CC of 0.539. At 30 min, colocalisation between the two proteins is seen throughout the cytoplasm and the along the nuclear membrane with a CC of 0.626. For the hPDLSCs-*GRP78* grown in OD media, colocalisation is seen at both 15 and 30 min like the control; however, at 15 min (CC 0.526), the colocalisation of the proteins occurred within the cytoplasm. In the OD conditions, greater colocalisation (CC 0.648) was observed at 30 min around the nuclear membrane and within the cytoplasm, suggesting that the differentiation medium allows for faster trafficking of rDMP1-GRP78 complex to the nucleus than the control. Fig. 1b show the presence of mesenchymal stem cell marker STRO-1 in hPDLSCs.

### Bioinformatic analysis of RNA-Seq data using DAVID and IPA to identify the transcriptome profile of associated osteogenic and angiogenic genotype changes with *GRP78* overexpression

**Fig. 2.**
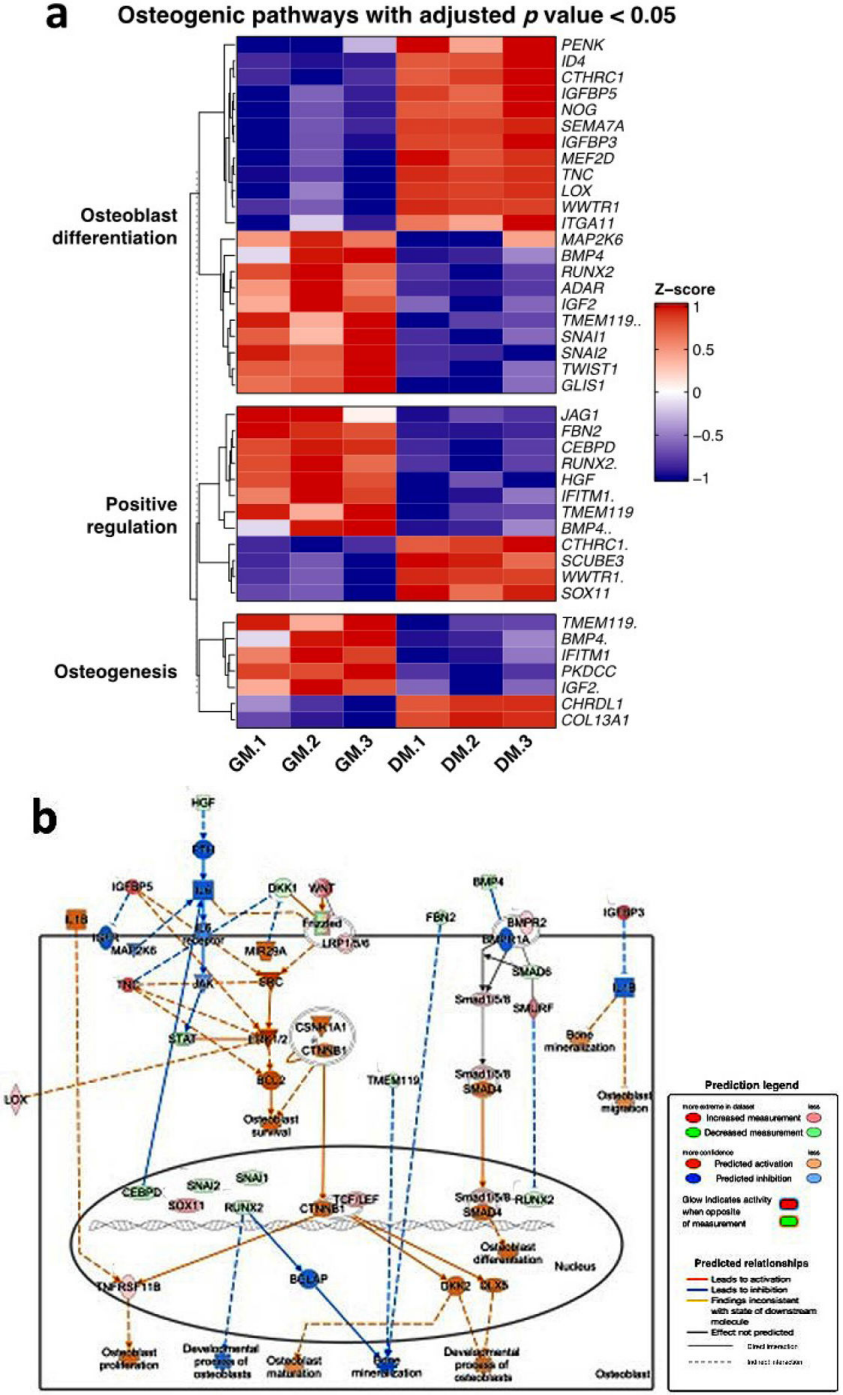
Osteogenic pathway analysis of hPDLSCs-GRP78 using DAVID and IPA. (**a**) RNA sequencing data from hPDLSCs-*GRP78* cultured under normal conditions (control) and under OD conditions for 2 d. Heatmap and hierarchical clustering of genes involved in osteogenesis development and function was generated using the bioinformatics program DAVID. The expression levels of genes are indicated by the colour bar. Red colour indicates increased expression whereas blue indicates the decreased expression as compared to control. (**b**) The analysed data was then designed on a pathway for osteogenesis to analyse the gene network being up or down regulated in the pathway using IPA. The brightness of colour is related to the fold change of differentially expressed gene and darker the colour the higher the fold change.

**Fig. 3.**
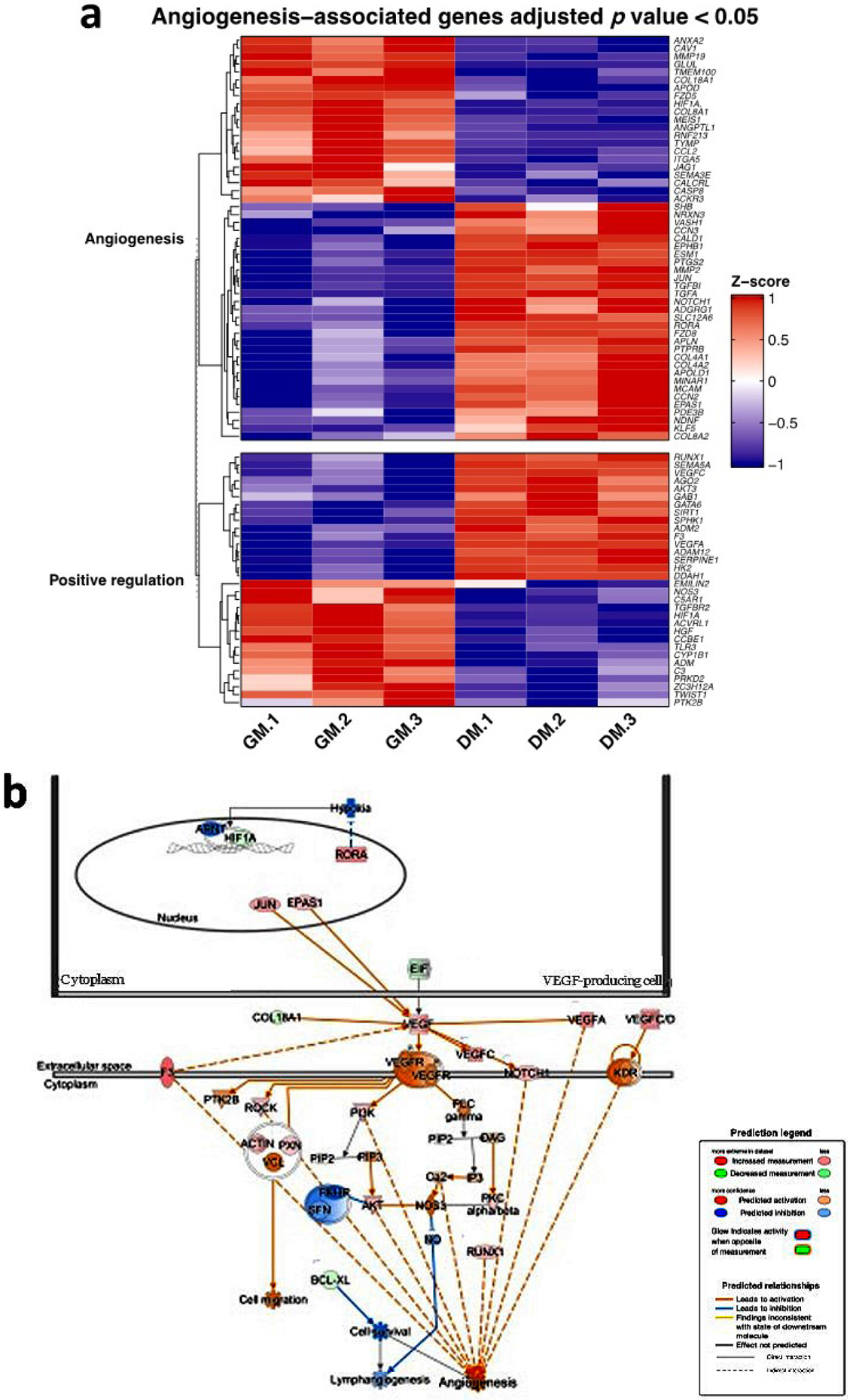
Angiogenic pathway analysis of hPDLSCs-*GRP78* using DAVID and IPA. (**a**) RNA sequencing data from control hPDLSCs-*GRP78* cultured under normal conditions (control) and under OD conditions for 2 d. Heatmap and hierarchical clustering of genes involved in angiogenesis development and function was generated using the bioinformatics program DAVID. The expression levels of genes were indicated by the colour bar. Red colour indicates increased expression whereas blue indicates the decreased expression as compared to control. (**b**) The analysed data was then designed on a pathway for angiogenesis to analyse the gene network being up or down regulated in the pathway using IPA. The brightness of colour is related to the fold change of differentially expressed gene and darker the colour the higher the fold change.

In order to understand the molecular mechanisms underlying GRP78-mediated osteogenic and vasculogenic differentiation, RNAseq data was analysed by bioinformatics analysis for gene expression profiles. DAVID bioinformatics and IPA, an advanced bioinformatic software program that can analyse the gene expression pattern using a scientific literature-based database, was used. Analysis of the heatmap with hierarchical clustering showed the gene expression profiles specific for osteogenesis. The heatmap depicted in Fig. 2a suggested that positive regulators of osteoblast differentiation and osteogenesis genes were down regulated in the controls and up regulated with differentiation. Notable genes were *HGF2, Smad5, CCN1, ECM1* and *Col13A1*. Results from the RNA sequencing are also depicted in a pathway format from IPA in Fig. 3b. The IPA software can take the analysed datasets and populate the up-and downregulated genes from the OD data compared to the control hPDLSCs-*GRP78* and the differentiated hPDLSCs-*GRP78*. In the pathway from IPA specific module, “the role of osteoblasts, osteoclasts in rheumatoid arthritis” the osteogenic specific genes were observed as shown in Fig. 2b. Several genes and signalling pathways responsible for osteoblast survival, osteoblast differentiation and osteoblast function were upregulated. The IPA software takes into account published data and relationships between molecules to grow and add onto the data as shown. The global view of angiogenic markers in the heatmap (Fig. 3a) show several genes responsible for angiogenesis such as *HSPG2*, members of the Wnt pathway such as *RORA* and *FRZ8* and other genes such as *MMP2, TGFβ, EPHB1, AMOTL1* and *2*. In the genes responsible for sprouting angiogenesis, *VEGFA, B, C, Notch 1, ANGPT, THBS1* were upregulated in cells overexpressing *GRP78* and in the presence of differentiation media. IPA analysis confirmed the upregulation of several pathways responsible for angiogenesis such as migration of cells, invasion of cells, blood vessel maturation, cell survival, iron ion transport and RBC production (Fig. 3b). Overall, the results suggest that cells overexpressing *GRP78* are capable of expressing markers promoting angiogenesis and osteogenesis when undergoing differentiation.

### Overexpression and silencing of *GRP78* influences osteogenesis and angiogenesis

**Fig. 4.**
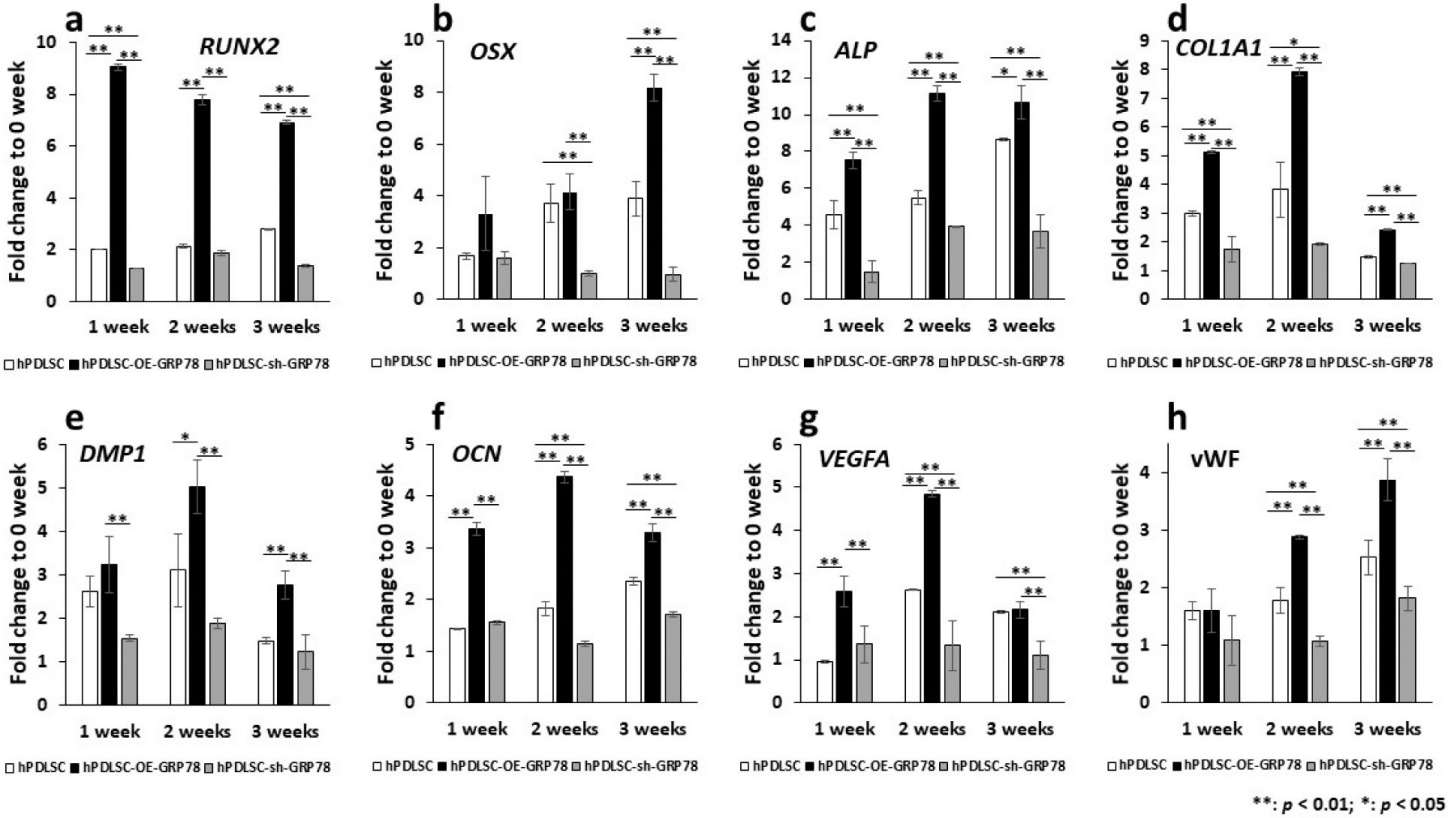
Gene expression analysis of osteogenic and angiogenic markers under osteogenic conditions. Total RNA was isolated from hPDLSCs, hPDLSCs-OE-*GRP78*, and hPDLSCs-sh-*GRP78* in osteogenic condition for 1, 2, and 3 weeks. The results were normalised to 1 from 0 week. Fold change was calculated with respect to the control that was normalised as 1. Values are the mean ± standard deviation of triplicate samples. Comparisons were performed using one-way *ANOVA* followed by Tukey’s multiple comparison test. A statistically significant difference is denoted with * *p* < 0.05 *vs*. control; ** *p* < 001.

In order to assess the influence of GRP78 on the cellular differentiation process, hPDLSCs, hPDLSCs-OE *GRP78*, and the hPDLSCs-sh-*GRP78* were cultured in OD media and the total RNA was isolated after 1, 2, and 3 weeks. RT-PCR results (Fig. 4) show that the periodontal stem cells overexpressing GRP78 exhibit an upregulation in the “early” osteogenic genes *RUNX2, OSX* at 1 week while *OSX* expression progressively increased until 3 weeks. *ALP* expression levels in the control hPDLSCs increased from 1-3 weeks; however, its expression with GRP78 overexpression resulted in a 10-fold increase. The predominant bone matrix protein, COL1A1 was highly expressed at 2 weeks compared to the other cell types. Regulatory proteins involved in mineralisation such as DMP1 and OCN increased at 1 and 2 weeks and decreased at 3 weeks. Silencing *GRP78* resulted in lower expression levels of RUNX2, OSX, ALP, COL1A1, DMP1 and OCN in all 3 cell types. Validation of angiogenic markers show upregulation of VEGFA, the key mediator of vasculogenesis at 1 and 2 weeks and silencing *GRP78* attenuated its expression levels, while vWF increased for 3 weeks with *GRP78* overexpression (Fig. 4).

### Effect of DMP1 and differentiation media on the osteogenic and angiogenic potential of GRP78-overexpressing cells

**Fig. 5.**
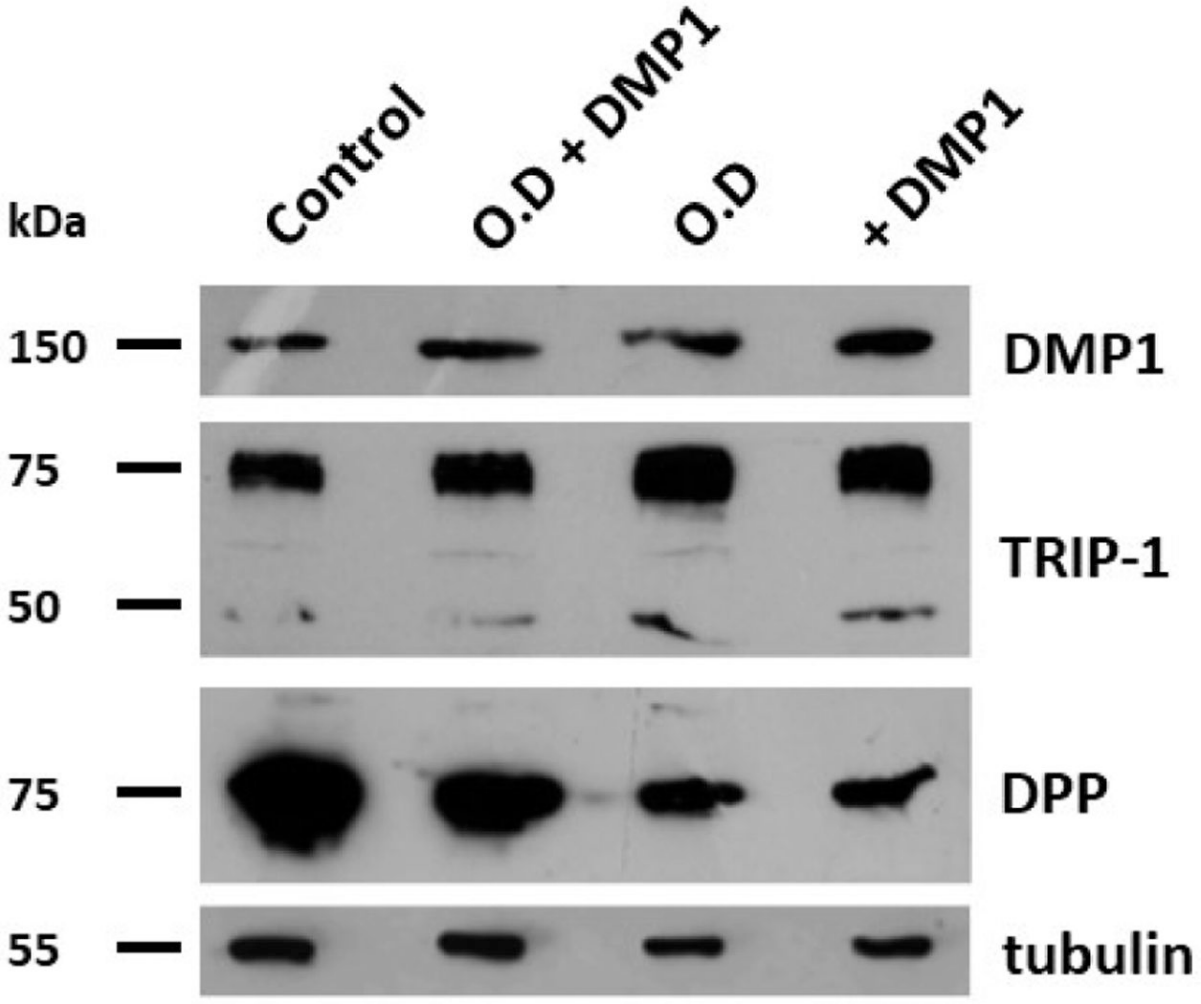
Protein expression of osteogenic and angiogenic markers. (**a**) hPDLSCs-*GRP78* were grown under normal growth conditions (control), OD, normal growth condition with rDMP1 (+DMP1), and OD conditions with rDMP1 (OD + DMP1) conditions until 70-80 % confluence. The conditions with rDMP1 were stimulated with DMP1 for 24 h prior to harvesting. Western Blot Analysis for antibodies against DMP1, TRIP-1, and DPP. Loading was confirmed with tubulin.

GRP78 interacts with DMP1 and facilitates its internalisation. Therefore, whether DMP1 stimulation on cells overexpressing GRP78 would promote cellular differentiation was assessed. For this, periodontal ligament stem cells overexpressing GRP78 were treated with both normal media and OD media with and without DMP1, to examine its influence on osteogenic and angiogenic differentiation potential. Western blotting data presented in Fig. 5 shows higher levels of DMP1 expression when GRP78 overexpressing cells were cultured in differentiation media with DMP1 stimulation. TRIP-1, a protein involved in angiogenesis and matrix mineralisation, was upregulated under all conditions when compared with the control; however, the OD condition and DMP1 stimulation had a higher influence on TRIP1 expression. DPP a key non collagenous protein in the bone and dentine matrix is lower in the OD conditions stimulated with rDMP1 than the control. This suggests that the differentiation conditions may trigger the transport of DPP to the secretome.

### GRP78 promotes mineralisation of the extracellular matrix

**Fig. 6.**
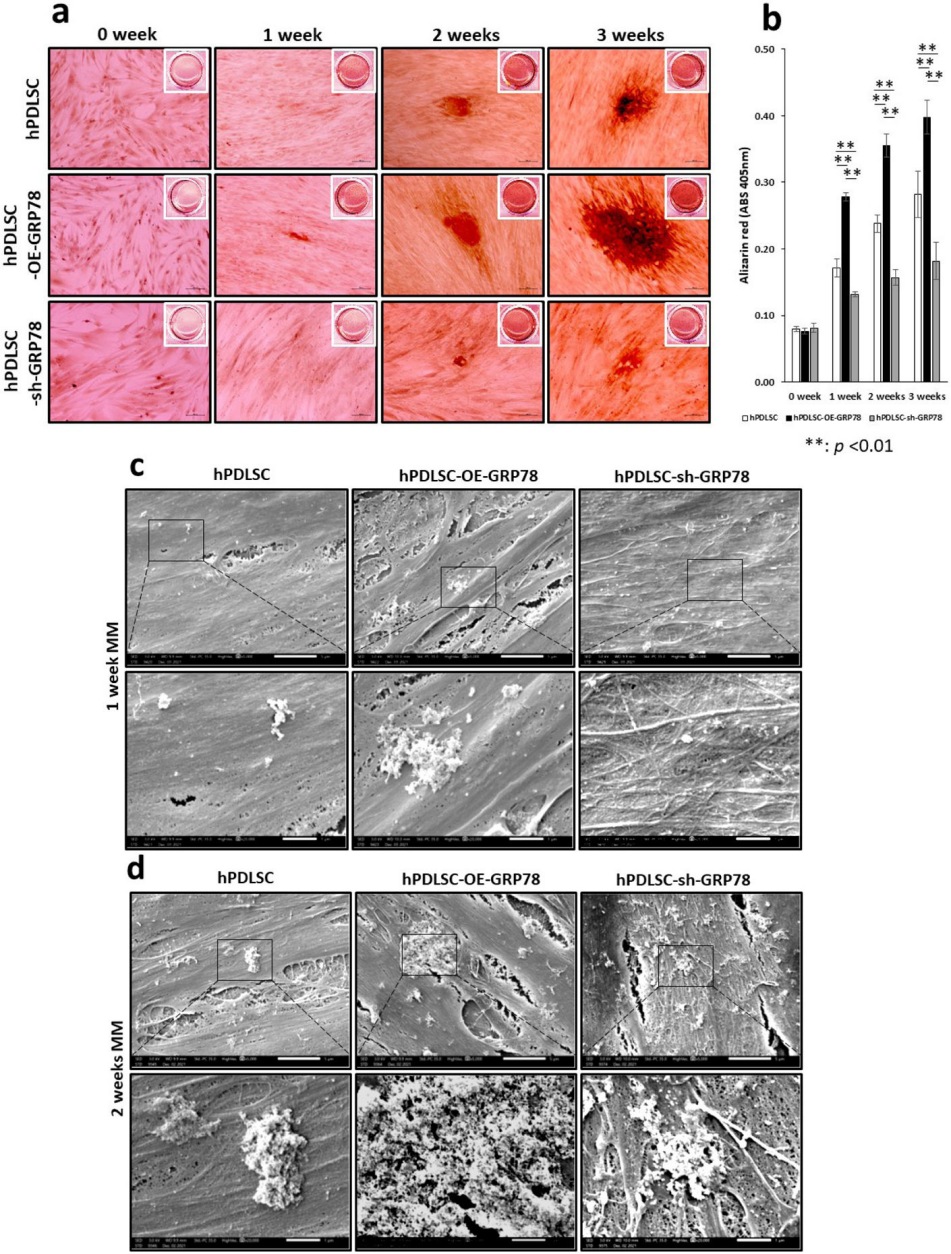
GRP78 promotes matrix mineralisation as determined by alizarin red staining and electron microscopy. hPDLSCs, hPDLSCs-OE-*GRP78*, and hPDLSCs-sh-*GPP78* cells were grown in MM for 1, 2 and 3 weeks. Mineralised nodules containing calcium were visualised using alizarin red staining. (**a**) Higher magnification of the images shows calcium deposits in the mineralisation cultures at various time points. (**b**) Quantitative measurement of calcium deposition was determined by measuring the absorbance of the eluted alizarin red stain at 562 nm on a multiplate reader using a standard calcium curve. Statistically significant differences are indicated at 1, 2 and 3 weeks ** *p* < 0.01. Scale bars = 100 μm.

To demonstrate the role of GRP78 in mineralised matrix formation, alizarin red staining was performed. Results in Fig. 6a show increased mineral deposits at 1, 2 and 3 weeks in the hPDLSC-OE-*GRP78* group when compared with the control and silencing of *GRP78*. SEM images presented in Figs. 6b and 6c show increased mineral deposits with GRP78 overexpression.

### GRP78 overexpression stimulates the expression of osteogenic and angiogenic markers in an *in vivo* subcutaneous implant model

**Fig. 7.**
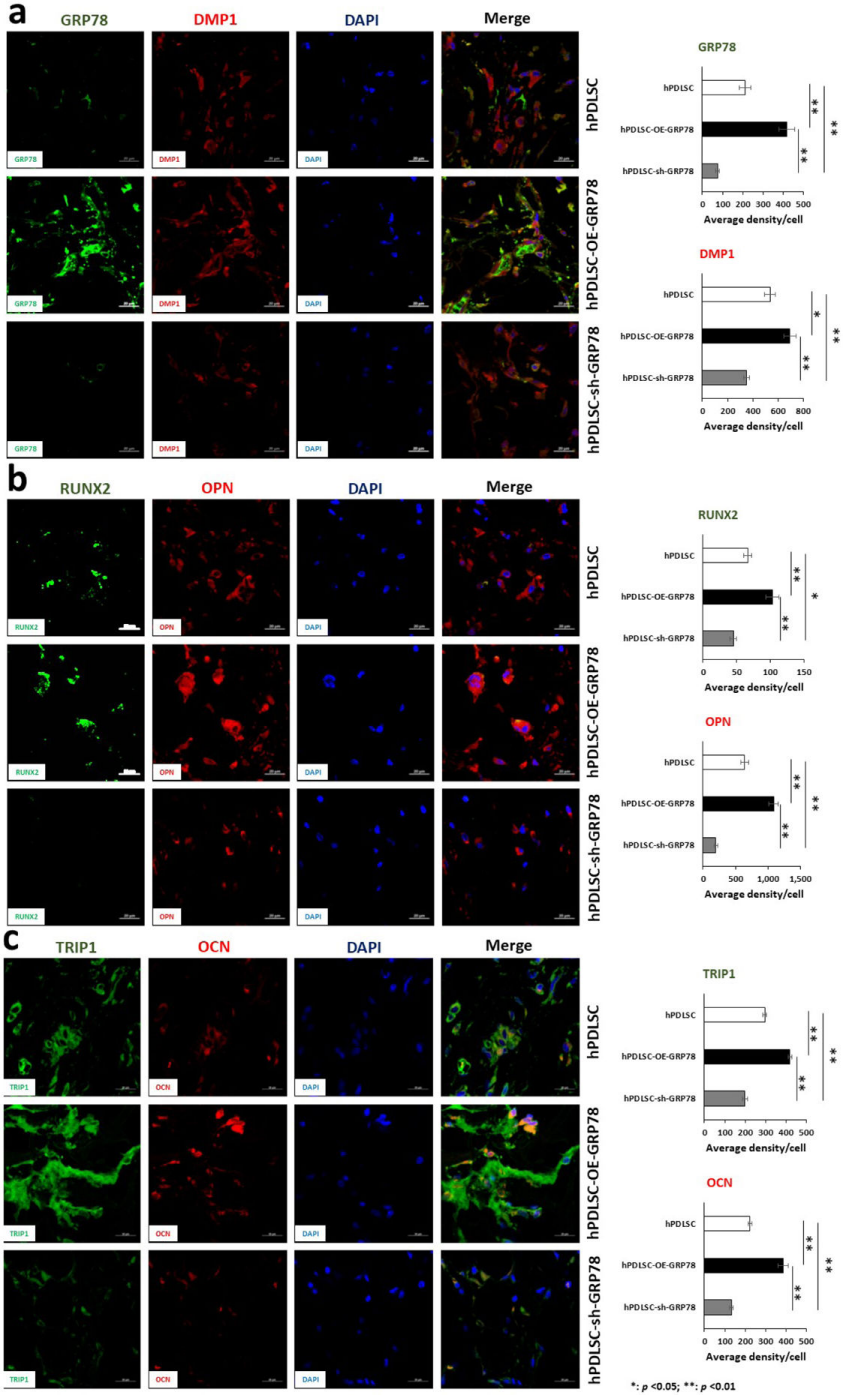
*In vivo* analysis of explants for osteogenic markers. Representative confocal micrographs showing the immunolocalisation of key osteogenic genes. The 3 groups were control hPDLSCs, hPDLSCs-*GRP78*, or hPDLSCs-sh-*GRP78* on the collagen scaffold. The explants were probed for (**a**) *GRP78* (FITC), *FL-DMP1* (TRITC), (**b**) *RUNX2* (FITC), *OPN* (TRITC), and (c) *TRIP1* (FITC), and *OCN* (TRITC) with DAPI being the nuclear stain. Microscopy was performed at the UIC Microscopy Core using Zeiss Meta 710 Confocal Microscopy. Scale bars = 10 μm.

**Fig. 8.**
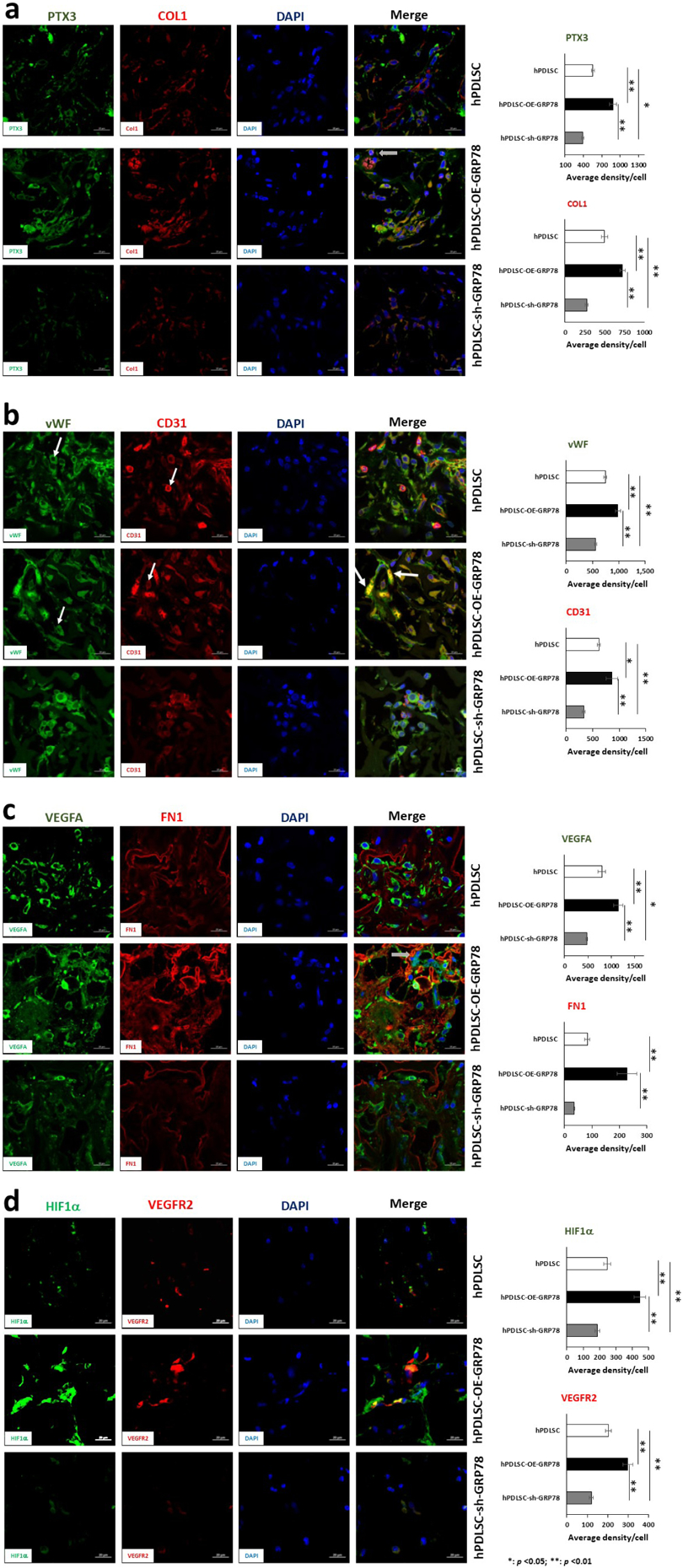
Analysis of explants for angiogenic markers. Representative confocal micrographs showing the immunolocalisation of Angiogenic markers. The three groups were control hPDLSCs, hPDLSCs-*GRP78* or hPDLSCs-sh-*GRP78* on the collagen scaffold. The explants were probed for (**a**) PTX3 (FITC), COL1a1 (TRITC), (**b**) vWF (FITC), CD31 (TRITC), (**c**) VEGFA (FITC), FN (TRITC), (**d**) HIF1α (FITC, and VEGFR2 (TRITC) with DAPI as the nuclear stain. Microscopy performed at the UIC Microscopy Core using Zeiss Meta 710 Confocal Microscopy. Arrows represent membrane localisation. Scale bars = 10 μm.

Whether GRP78 modulated osteogenesis and angiogenesis *in vivo* was explored next. For *in vivo* studies, control hPDLSCs, transgenic cell lines hPDLSCs-OE-*GRP78*, and hPDLSCs-sh-*GRP78* were seeded on collagen scaffolds and implanted into the dorsum of nude mice. The explants were retrieved after one month and examined for angiogenesis and osteogenesis by IHC. In Fig. 7 and 8, the osteogenic and angiogenic markers show increased expression of important markers in the PDLSC-OE-*GRP78* group and abrogated with silencing *GRP78*. DMP1, a key regulatory protein in mineralisation showed significantly increased expression in the OE-*GRP78* group *versus* the control. Knockdown of *GRP78* demonstrated decreased expression of DMP1 (Fig. 7a). RUNX2, an early bone formation transcription factor is expressed in both the control and GRP78 overexpressing cells but downregulated in silenced *GRP78* cells. Interestingly, OPN a regulator of mineralisation was highly expressed with GRP78 overexpression (Fig. 7b). TRIP1 and OCN, important proteins in matrix mineralisation and angiogenesis, were similarly upregulated (Fig. 7c). Among the angiogenic markers, *PTX3* (Greggi et al., 2021; Presta et al., 2018) was upregulated with *GRP78* and downregulated with *GRP78* knocked down (Fig. 8a). The major extracellular matrix protein in bone and dentine, COL1A1, showed expression levels higher than the control (Fig. 8a). Key angiogenic proteins VEGFA and its receptor VEGFR2 and FN were upregulated with GRP78 (Fig. 8b,c). HIF1α, a key transcription factor for the induction of VEGF, is upregulated with GRP78 overexpression when compared with the control. However, when *GRP78* is knocked down the expression level is attenuated (Fig. 8c). Interestingly, HIF1α is localised to the nuclear membrane and cytoplasm in the *GRP78* group. Runx2 is also required for the stabilisation of HIF1α and is necessary for the activation of VEGF (Kwon et al., 2011; Lee et al., 2012).

### Histological evaluation of the explant tissue

**Fig. 9.**
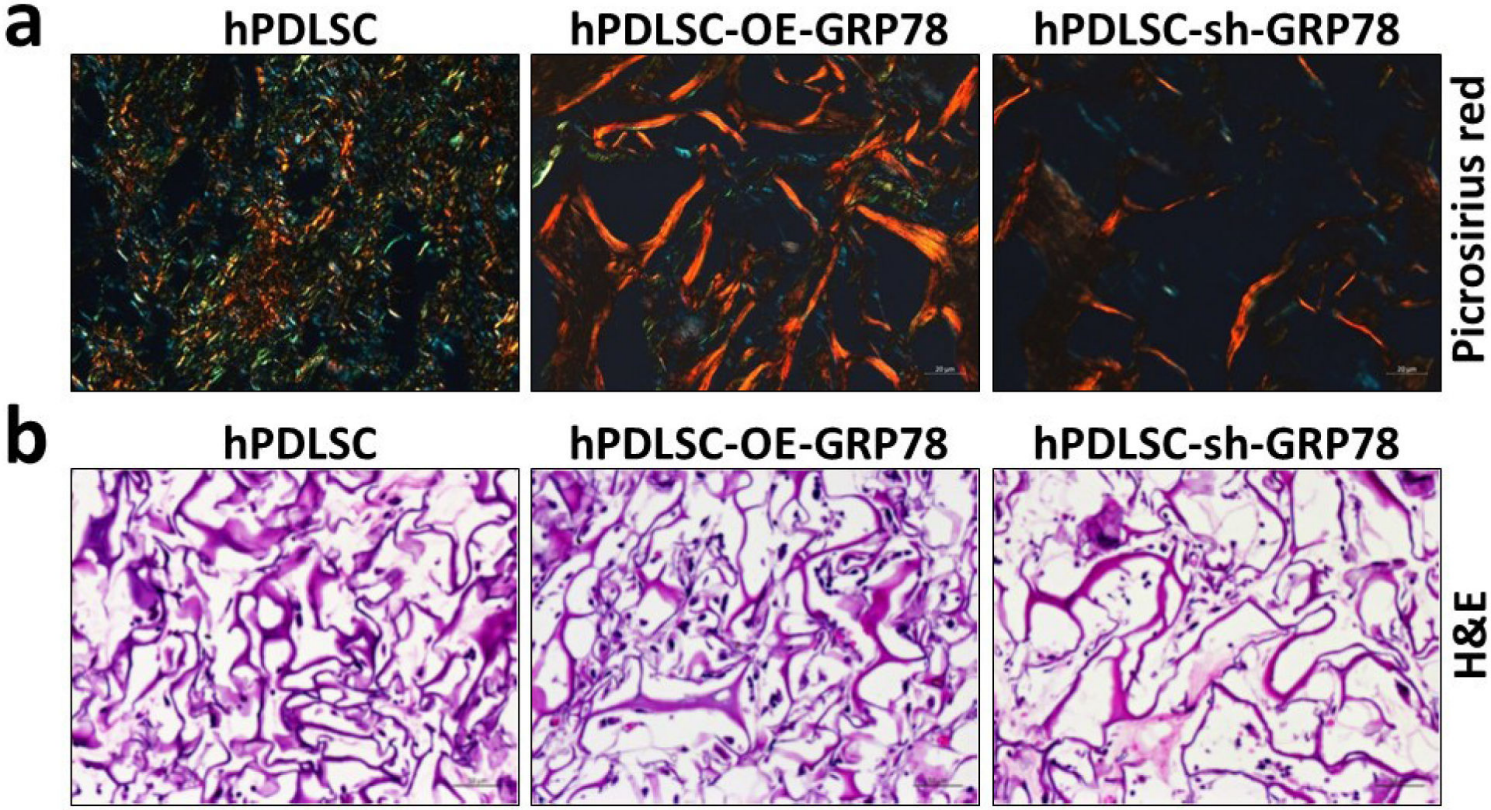
Analysis of explants to demonstrate morphology of the collagen fibrils and tissue architecture. (**a**) The explants were subjected to picrosirius red staining. The stain was analysed using polarised light to determine the morphology of the collagen fibrils for the control hPDLSCs scaffold, hPDLSCs-*GRP78* scaffold, and the hPDLSCs-sh-*GRP78* scaffold. Scale Bar = 20 μm. (**b**) The hPDLSCs scaffold, hPDLSCs-*GRP78* scaffold, and the hPDLSCs-sh-*GRP78* scaffold stained with H&E staining. Scale bar = 50 μm.

Picrosirius red staining of the matrix from the control hPDLSCs under polarised light showed collagen fibrils with a green birefringent colour which is indicative of collagen fibrils with normal physiological diameter while the collagen fibrils deposited with GRP78 overexpression showed yellowish orange birefringent colours, indicative of thicker collagen fibrils, which are densely packed. In contrast, silencing *GRP78* resulted in a matrix containing fewer and thinner collagen fibrils with red and blue birefringence (Fig. 9a). The H&E stain (Fig. 9b) demonstrated increased cellular density within the matrix of OE-*GRP78* cells when compared with the control and *GRP78* knocked-down cells.

## Discussion

Almost half the adult population in the United States suffers from periodontal disease, a progressive disease that is a result of attachment loss of the tooth to the underlying alveolar bone. The advancement of periodontal disease was classified by the AAP in 2017 to give both staging and grading of the disease. Risk factors like smoking, hypertension, and diabetes can accelerate periodontitis leading to vertical and horizontal bone loss (Caton et al., 2018). The current strategies to help periodontal disease in patients includes mechanical debridement of the affected cementum and scaling the calculus to reduce future attachment loss (Lazar et al., 2016). Prevention of attachment loss is the main goal for periodontal disease patients; however, strategies to regenerate the lost bone need to be evaluated to help the patient have better success in combating tooth loss in the future. Bone regeneration in dentistry has followed different avenues and strategies. In the current study, the goal was to promote alveolar bone regeneration by utilising site-specific stem cells from the periodontium along with GRP78 as a therapeutic molecule to promote OD of PDLSCs with mineralised matrix formation. Such a strategy can be used at sites of vertical defects or even horizontal bone loss due to periodontal disease or trauma to the periodontium.

Periodontal ligament stem cells are adult stem cells that are easily obtained from extracted teeth due to the availability of the retained periodontal ligament following extraction. These stem cells can differentiate into various lineages, such as fibroblasts, adipocytes and osteoblasts, and even neurogenic lineages (Zhu and Liang, 2015). Published reports have shown that PDLSCs are advantageous for use in regeneration due to their low immunogenicity because they do not express HLA-II or mount a T-cell response (Wang et al., 2020). As a result, of the low immunogenicity, these cells from different donors can be used for tissue regeneration. PDLSCs are capable of OD by expressing increased levels of RUNX2 – a transcription factor for osteoblast development – and ALP – an early marker for osteoblast differentiation – when cultured in differentiation media for 7 to 14 d (Merkel et al., 2019). In this study, the potential of hPDLSCs to overexpress GRP78, and promote osteogenesis and angiogenesis – thereby aiding functional bone regeneration – was demonstrated.

The SIBLING family of proteins are integral to the mineralisation of bone and dentine, especially DMP1 and DPP. Mutations in DMP1 have been associated with diseases such as osteomalacia and rickets due to altered phosphate metabolism (Feng et al., 2006; Zhao et al., 2011). A knockout DMP1 mouse model additionally shows altered PDL structure and morphology suggesting that DMP1 not only has an important regulatory role in mineralised tissues, but also in the surrounding tissues of the periodontium (Ren et al., 2016). Published reports have shown that DMP1 functions both intracellularly as well as in the extracellular matrix and is a transcriptional regulator for osteoblast differentiation (Jacob et al., 2014). Previous research linked a functional relationship between DMP1 and GRP78, an ER chaperone that activates the UPR and aids in cell homeostasis. Although its major function is to act within the ER, GRP78 has numerous functions outside the ER that shows its functional diversity. The presence of GRP78 on the cell surface was completely unexpected and studies by Ni et al. (2011) demonstrate that GRP78 acts as a viral receptor at the plasma membrane. *In vitro* mass spectroscopy assays and TIRF microscopy studies have also shown that GRP78 and DMP1 interact at the plasma membrane during differentiation of preosteoblasts and preodontoblasts (Ravindran et al., 2008). Furthermore, the mechanism of vesicular trafficking of the DMP1-GRP78 complex and subsequent transport of DMP1 to the nucleus has been determined (Merkel et al., 2019). However, the mechanism by which DMP1 is released from the endosomes to the cytoplasm is yet to be determined.

In the current study, a stable cell line of hPDLSCs overexpressing GRP78 was first established to demonstrate its role in mineralisation. Stimulation of the cells with DMP1 demonstrated rapid internalisation mediated by GRP78. Increased colocalisation of the two proteins was observed in the cytoplasm under OD conditions. Interaction of GRP78 with DMP1 and the subsequent intracellular transport of the receptor-ligand complex, culminating with the transfer of DMP1 to the nucleus, might be essential for the OD process.

The expression of angiogenic and osteogenic genes was then examined, as they are crucial for the development of functional alveolar bone. Published reports show that under OD conditions, hPDLSCs express early bone markers such as ALP and RUNX2 (Zhu and Liang, 2015). In the current study, that overexpression of GRP78 promoted the expression of early osteogenic markers such as Runx2 and ALP was demonstrated. Runx2 is the master transcription factor for osteoblast differentiation and ALP provides the phosphate source for matrix mineralisation. Other early bone markers that were upregulated include mRNA for COL1A1 and OSX, which are also classified as markers for early osteoblast differentiation (Shetty et al., 2016). Further, regulatory proteins involved in mineralisation such as DMP1 and DPP increased with GRP78 overexpression. DPP protein levels showed decreased expression levels with GRP78 overexpression in the presence of differentiation media and DMP1 treatment. This is attributed to the transport of DPP to the ECM in extracellular vesicles during differentiation (Zhang et al., 2014). In the matrix, DPP binds to collagen and facilitates hydroxyapatite nucleation and growth picrosirius red staining, along with polarised light microscopy, revealed the arrangement of collagen fibrils in the matrix. Birefringence in orange-red colours of the thick collagen fibrils in GRP78 overexpressing cells, demonstrated that GRP78 facilitates the assembly of mature closely-packed fibrils, which is essential for mineral deposition. Collagen fibrils with shades of green-yellow, as observed in *GRP78*-silenced cells, depict immature procollagen.

Interestingly PDLSCs overexpressing GRP78 cultured under normal growth conditions showed higher gene expression of *Runx2* at 1 and 2 weeks, suggesting that GRP78 overexpression was sufficient to initiate the transformation of PDLSCs into osteoblasts. On the other hand, RNA-seq data show that PDLSCs overexpressing GRP78, cultured under differentiation conditions for 2 d, downregulates *Runx2* expression but was highly expressed in the control PDLSCs overexpressing GRP78 – which correlated with the gene expression data obtained by RT-PCR. Although qPCR and RNA-seq are both used to measure gene expression, the unit of measurement is different. The fold change should not be expected to be the same for the two methods. This is due to the normalisation process of data analysis. The initial upregulation of Runx2 expression in the differentiation media followed by lower expression with differentiation corroborates well with published data that suggests Runx2 as the “master” transcription factor for the initiation of osteoblastogenesis and is downregulated in mature osteoblasts (Komori, 2010).

The terminal differentiation stage of osteoblasts is the formation of a mineralised matrix. In this study, alizarin red staining of hPDLSCs overexpressing GRP78 showed more calcium deposits when compared with the *GRP78* knocked-down cells. SEM analysis further confirmed the presence of mineral deposits in the ECM. These findings imply that GRP78 can aid in the OD of PDLSCs and mineralised matrix formation. Identifying GRP78 as a promoter of mineralisation would be advantageous in tissue regeneration applications. Bioinformatics analysis of the gene network data suggest that key signalling pathways in OD – such as the AKT, BMP and TGFβ pathways – are significantly upregulated, suggesting the downstream signalling events promoting the transcription of genes that are involved in osteogenesis. RNAseq data, further confirmed the potential function of GRP78 in osteogenesis with upregulation of genes such as *DDR2*, which stimulates osteoblast differentiation (Ge et al., 2018). Matricellular protein CCN1 was upregulated, and this protein is known to modulate mature osteoblast and osteocyte function to regulate bone mass through angiogenesis as well as by modulating Wnt signalling (Zhao et al., 2018). Upregulation of FBX05 transcripts was interesting, as this protein is known to promote migration and OD of hPDLSCs (Liu et al., 2018).

Another key factor in the development of bone is vasculogenesis, the formation of new blood vessels. Nutrients and growth factors are necessary for developing bone, and blood vessels allow the transport of nutrients and removal of metabolic wastes. Markers for angiogenic activity include VEGFA, which is the main signalling molecule associated with the induction of angioblasts to form new vasculature (Hu and Olsen, 2016). Examination of the gene network for angiogenesis by IPA show upregulation of HIF1α and VEGFA. Under hypoxic or low oxygen conditions, *HIF1α* is activated to promote vasculogenesis. In conditions where *GRP78* is silenced, downregulation of both *VEGFA* and *HIF1α* was observed confirming that GRP78 has a functional role in vasculogenesis (Kuo et al., 2013). Overall, the data suggest an intimate role between GRP78 and angiogenesis; however, future research is required to elucidate the signalling pathway for activation of angiogenesis.

Tissue regeneration is the process of restoring the tissue to its original architecture. Currently in the periodontium, guided bone regeneration with matrix proteins or enamel-matrix derivatives are the main source of potential treatment (Tonelli et al., 2011). In order to assess if GRP78 could be utilised as a therapeutic molecule for repair of the periodontium, the *in vivo* function of GRP78 was assessed by subcutaneous implantation of a 3D scaffold with genetically modified PDLSCs. Immunohistochemical analysis of the explants stained for GRP78, showed increased expression in the matrix of overexpressing cells with reduced expression in the knockdown. This implies that cells are genetically modified, and the observed phenotype is due to the presence or absence of *GRP78* and its associated signalling pathways. Functionality of the differentiated cells toward osteogenic lineage was examined by the expression levels of DMP1, RUNX2, OPN, OCN, COL1A and TRIP-1, which were highly expressed in comparison to the control and knockdown cells. TRIP-1 has been identified as a modulator of matrix mineralisation and angiogenesis (Chen et al., 2018; Ramachandran et al., 2016). Analysis of angiogenic markers, showed higher levels of VEGFA, PTX3, vWF, CD31, HIF1α, FN and VEGFR2. Silencing *GRP78* attenuated their expression levels. This is consistent with published data suggesting a knockdown of *GRP78* in NPC cells also shows a decrease in VEGFA and Ang2 (Fu et al., 2016). Summarising, the *in vivo* subcutaneous implantation model confirmed a functional role of GRP78 in angiogenesis and osteogenesis.

The results of the current study imply that GRP78 could be developed as a novel therapeutic target for tissue repair and regeneration of the diseased periodontium. Strategies to deliver GRP78 on collagen sponge could be envisaged as an ideal therapeutic approach within the periodontium to restore defects in alveolar bone. Future studies need to be done with utilisation of other vehicles for the delivery of GRP78 to the periodontium. This is the first promising report with respect to the use of GRP78 as a therapeutic target in matrix mineralisation and vascularisation.

## List of Abbreviations

3D: 3-dimensional
AAP: American Academy of Periodontology
AKT: AKT serine/threonine kinase1
ALP: alkaline phosphatase
AMOTL1: angiomotin-like1
AMOTL2: angiomotin-like2
Ang2: angiogenin 2
ANGPT: angiopoietin
ANOVA: analysis of variance
ATF6: activating transcription factor 6
BMP: bone morphogenetic protein
BSA: bovine serum albumin
CC: correlation coefficient
CCN1: cellular communication network factor 1
cDNA: complementary DNA
CD31: cluster of differentiation 31
COL1A1: collagen type 1 alpha 1 chain
Col13A1: collagen typeXIII alpha 1 chain
CPM: counts per million
DAPI: 4', 6-diamidino-2-phenylindole
DAVID: database for annotation, visualisation,and integration discovery
DDR2: discoidin receptor 2
DMP1: dentine -matrix protein-1
DPP: dentine phosphoprotein
ECM: extracellular matrix
ECM1: extracellular matrix protein 1
EPHB1: ephrin B1
ER: endoplasmic reticulum
FBS: foetal bovine serum
FBX05: F-box protein 5
FDR: false discovery rate
FITC: fluorescein isothiocyanate
FL-DMP1: full length dentine matrix protein 1
FN: fibronectin
FRZ8: frizzled-8
GAPDH: glyceraldehyde-3-phosphate dehydrogenase
GFP: green fluorescent protein
GRP78: glucose-regulated protein-78
H&E: haematoxylin and eosin
HGF2: hereditary gingival fibromatosis type2
HIF1α: hypoxia inducible factor
HLA-II: human leukocyte antigen class II
hPDLSCs: human periodontal ligament stem cells
HRP: horseradish peroxidase
HSPG2: heparan sulphate proteoglycan2
IHC: immunohistochemistry
IPA: ingenuity pathway analysis
IRE1: inositol-requiring transmembrane kinase1
JACoP: just another co-localisation plugin
MM: mineralisation media
MMP2: matrix metalooproteinase 2
Notch 1: neurogenic locus notch homologue protein 1
NPC: nasopharyngeal carcinoma
OCN: osteocalcin
OD: osteogenic differentiation
OE: overexpression
OPN: osteopontin
OSX: osterix
PBS: phosphate-buffered saline
PCA: principal component analysis
PCC: Pearson’s coefficient of colocalisation
pCDH: lentiviral vector
PCR: polymerase chain reaction
PDL: periodontal ligament
PDLSCs: periodontal ligament stem cells
PERK: protein-kinase like ER kinase
PTX3: pentraxin 3
Rab: ras-associated binding protein
RBC: red blood cells
rDMP1: recombinant dentine matrix protein 1
RIPA: radioimmunoprecipitation buffer
RNA-Seq: RNA sequencing
RORA: RAR related orphan receptor A
RUNX2: runt-related transcription factor 2
SDS: sodium dodecyl sulphate
SEM: scanning electron microscope
sh: small hairpin
SIBLING: small integrin-binding ligand, N-linked glycoprotein
Smad5: mothers against decapentaplegic homolog 5
STRO-1: mesenchyme marker
TGFβ: transforming growth factor β
THBS1: thrombospondin 1
TRIP-1: TGFβ receptor type II interacting protein-1
TIRF: total internal reflection microscope
TRITC: tetramethylrhodamine
UIC: University of Illinois Chicago
UPR: unfolded protein response
VEGF: vascular endothelial growth factor
VEGFA: VEGF-a
VEGFB: VEGF-b
VEGFC: VEGF-c
VEGFR2: VEGF-receptor2
vWF: von Willebrand factor
Wnt: wingless-related integration site
αMEM: alpha minimum essential medium
